# The BASE-Program—A Multidimensional Approach for Health Promotion in Companies

**DOI:** 10.3390/healthcare4040091

**Published:** 2016-12-08

**Authors:** Bettina Wollesen, Josefine Menzel, Heiko Lex, Klaus Mattes

**Affiliations:** 1 Department of Human Movement Science, University of Hamburg, 20148 Hamburg, Germany; josefine.menzel@uni-hamburg.de (J.M.); klaus.mattes@uni-hamburg.de (K.M.); 2 Institute of Sport Science, University of Rostock, 18057 Rostock, Germany; heiko.lex@uni-rostock.de

**Keywords:** occupational health promotion, prevention, assessment, ergonomics, behaviour modification, intervention, motivation, cognitive representation

## Abstract

Multidimensional assessments for conducting interventions are needed to achieve positive health effects within companies. BASE is an acronym, consisting of B = “Bedarfsbestimmung” (requirements); A = “Arbeitsplatzorganisation” (organisation of work); S = “Schulung des belastungsverträglichen Alltagshandelns” (coaching preventive behaviour at work); E = “Eigenverantwortung und Selbstwirksamkeit” (self-responsibility and self-efficacy). It is a prevention program designed to avoid and reduce work-related musculoskeletal diseases. It was developed to support prevention strategies within companies. It comprises aspects of health protection, ergonomics, exercise and self-efficacy. A comprehensive assessment will identify strain e.g., musculoskeletal discomforts due to body positions or psychological stress. Moreover, the general health status, preferences and barriers for participating in health promotion programs are evaluated. This analysis leads to practical and goal-oriented recommendations and interventions which suit the needs of companies and employees. These are executed onsite in real workplace situations and involve the introduction of first-hand experience in behavioural change. Therefore, this practical approach enhances the employees’ acceptance and self-efficacy for health promotion. This can result in long-term health promoting behaviour. This article presents the outcome and sustainability effects of BASE in three different application fields (logistic, industrial and office workers).

## 1. Introduction

Due to the demographic change, altered working conditions and a prolonged working lifetime, the importance for occupational health promotion (OHP) has increased. The objective of OHP is the maintenance and promotion of the employees’ health, efficiency and productivity. Companies profit from OHP by increased economic and competitive ability, employees by positive effects on health and well-being [1].

In all European countries, musculoskeletal disorders are the most common cause of sick leave [2,3]. The results of different German health insurance reports show that musculoskeletal disorders have been one of the main reasons for an inability to work in recent years. Between 2011 and 2013, these disorders accounted for 21.3%–23.4% of sick leave days [4,5,6,7].

Due to the fact that distinct cause-and-effect relationships are still missing, there is consensus that specific occupational factors increase the risk of work related musculoskeletal disorders. Such factors comprise biomechanical overload [8], repetitive activities [9], lifting and carrying heavy stocks, positioning, ecological and psychosocial factors [10,11,12] as well as whole-body vibration [13]. From a biomechanical point of view, unfavourable body positions in combination with heavy weight lead to high stresses and strains of the musculoskeletal system [12]. Although underlying cause-and-effect relationships are not yet clarified [14], relationships between work-related musculoskeletal disorders in the shoulder and neck region and working on a computer [15], long sitting durations and less physical activity at the workplace [16] can be established. 

It is also worth mentioning that some musculoskeletal disorders including back or muscle pain may also be related to stress and work overload. Mental stresses like pressure of time, high intensity of labour, monotonous operational procedures as well as aspects of work organization may contribute to the onset of musculoskeletal disorders [17,18].

Nowadays, stress-related diseases like burnout are additional causes for absence from work that lead to an ever-increasing number of sick days. In Germany, sick leave owing to psychological disorders or mental distress increased from 6.6% to 13.1% between 2001 and 2010 [3]. 

Changes in work life, lifestyle and the increase in sick leave underline the importance of OHP-programs that focus on physical activity, mental well-being and a healthy work-life-balance or, rather, “life domain balance” [3]. Workplace health promotion programs (WHPP) can help achieve this life-domain balance. One specific challenge for companies engaged in health promotion is how to motivate employees to exercise and create a more active lifestyle in general. Committees of professional organizations like the World Health Organization (WHO) and the American College of Sports Medicine (ACSM) recommend at least 150–180 min of physical activity per week as well as 30 min of moderate activity during everyday life on five days a week [19]. 

Research history of WHPP with the goal of increasing physical activity and fitness has shown that the interventions have become increasingly specific and therefore the success of these programs has risen. Hence, a first review by Dishman et al. [20] failed to report benefits of increasing physical activity and fitness levels. Marshall [21] reported the success of those programs that considered aspects like the inclusion of the needs of the employees, the implementation of theories of behaviour change and a company network and potential trainers. This confirmed the findings of Proper et al. [22] who stated that the quality of implementation and its process is of particular importance for the success of workplace health promotion programs. A review by Taylor, Conner and Lawton [23] also pointed in this direction and underlined the importance of theory based programs. Beside the aspect of improved physical activity and fitness, other aspects of these health promotion programs also seem to be important. For example, Proper et al. [24] and Malik et al. [25] proposed that on site physical activity programs can support job satisfaction and reduce employee turnover. There is empirical proof that systematic interventions are able to improve physical and psychological health irrespective of age group. Andersen et al. [26] could demonstrate the effectiveness of specific weight training to reduce shoulder and neck pain in office workers. Oesch et al. [27] and van Tulder et al. [28] could provide evidence that work related weight training is effective in reducing work related musculoskeletal disorders. Besides weight training, several studies point out that ergonomic training, knowledge transfer [29] and stretching [30] are important instruments to prevent work related musculoskeletal disorders. Recent reviews focused on increasing productivity [31] and reducing obesity [32], but failed to provide evidence. 

Long-term implementation of these programs seems to be difficult, so positive effects are often observed immediately after intervention but sustainability has not been shown [33]. Rongen et al. [34] showed in a meta-analysis that the effectivity of interventions depends on the specific characteristics and design of the program. Interventions that take place on site of the workplace and in small groups are more effective. 

Following Healy et al. [35], interventions comprising both prevention of negative behaviour and conditions (e.g., work environment, work organization) are more effective. This finding is in line with the demands of New Public Health, that social influencing factors of health and disease should be taken into account to a greater extent and that interventions should be multidimensional as opposed to single behavioural prevention [36]. 

For initializing long-term behaviour modification and maintaining motivation of the employee, it is essential to emphasize the self-determination of the individual [37]. Accordingly, employees should be engaged in the planning and implementation of occupational health promotion programs. Wickström [38] recommends a “combined approach” for the successful implementation of occupational health promotion programs:

- The program ties in with the companies’ structures of the working conditions;
- The program considers the companies’ organizational and social environment;
- The program integrates the employees in the assessment of working health risks (participatory approach).

In addition, an effective health promotion program should include strengthening the degree of autonomy of the individual actions (e.g., by enhancement of decision-making authority) and realization of self-determination and personal responsibility [39].

The extension of safety at work through ergonomics, biomechanical assessment and movement instructions has proved to be a successful approach for companies [40]. Therefore, companies need programs that consider and integrate the specific demands, stresses and workloads of the employees to maintain and strengthen physical and psychological resources [41]. Consequently, multidimensional assessments for conducting interventions are needed to achieve positive health effects in companies.

**Figure 1:**
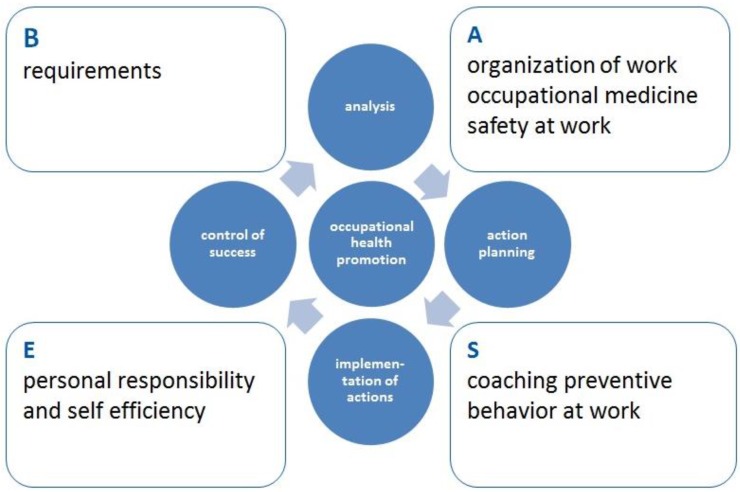
The basic structure of the BASE-program.

According to this theoretical framework, the BASE program (Figure 1) was designed as a prevention program to avoid and reduce work-related musculoskeletal diseases and mental stresses. BASE is a German acronym, consisting of B = “Bedarfsbestimmung” (requirements); A = “Arbeitsplatzorganisation” (organisation of work); S = “Schulung des belastungsverträglichen Alltagshandelns” (coaching preventive behaviour at work); E = “Eigenverantwortung und Selbstwirksamkeit” (self-responsibility and self-efficacy).

It comprises aspects of health protection, ergonomics, exercise and self-efficacy. A complex assessment identifies daily workloads, musculoskeletal discomforts, psychological stresses and general health states. Moreover, the companies’ infrastructure for health promotion as well as the organizational and social environment of the business (e.g., working hours, breaks, communication) are taken into consideration [10]. 

BASE is characterized by integration of the employees in each step of the assessment and implementation of the program. Specific wishes and barriers for health promotion programs are recorded to reach highest-possible participation quota. This extensive assessment leads to practical and goal-oriented recommendations and interventions. These are executed onsite in real workplace situations and involve the introduction of a first-hand experience in behaviour change. Kinesic behaviour during work is discussed and cognitively reflected upon with the workers to enhance the employee’s acceptance and self-efficacy for health promotion. This can result in long-term health promoting behaviour [10]. 

The training concept of BASE differs from conventional interventions for implementing an ergonomic and kinesic behaviour with a modified methodical format:

1. Daily activities are analysed within the initial assessment of requirements by observation of the workplace and/or video analyses. This helps to create functional load/exposure profiles and identify recurring movements of high load factor.
2. Based on the results of these analyses, adequate movement tasks are generated to train the coping with the usual demands of work. The introduction of a first-hand experience in behaviour change involves the movement experiences (titled the “AHA”-experience). This AHA experience is comprised of three components: (1) body awareness; (2) recognition of dysfunctional movements; and (3) understanding positive and negative behaviour in day-to-day working tasks. This serves as the initial step in preparing for the necessary change in automated movement behaviour.
3. The movement experiences are reflected on and internalized by discussion targeting the adaption of ergonomic motor execution to suit individual physical working conditions.
4. An explanation is provided about why the changed motor execution leads to reduced stresses and strains and how it can be implemented in day-to-day work. These considerations are made together by the exercise instructors and the employees.
5. The employees get the opportunity to put the new movement perceptions into practice by repeated solving of different movement tasks which they may encounter at work.
6. The principles of ergonomic motor behaviour are tested and reflected in different labour situations to facilitate a transfer to different actions in day-to-day work.

In contrast to previous interventions, this approach is based on an initial movement experience to spark the employees’ interest in the underlying theoretical background. This also helps to provide a better rapport with the employees from the onset. 

Since 2007, BASE has been implemented in 13 companies. The main research goals focussed on the implementation process, the feasibility of the intervention in different application fields and the acceptance of the whole program. This article presents the evaluation of the BASE program in three different application fields (logistic, industrial and office workers).

For all application fields, three stages will be reported:

1. Implementation of the BASE concept (stage one)
2. Evaluation of the outcome effects of the interventions (stage two)
3. Lasting effects and enhancing of health promotion (stage three)

Our hypothesises were, that (1) the implementation process of the program will influence the outcome effects; and (2) the whole approach will be able to gain lasting effects because of the integration of the multidimensional aspects of the WHPP.

## 2. Experimental Section

### 2.1. Application Fields and Goals

**Figure 2:**
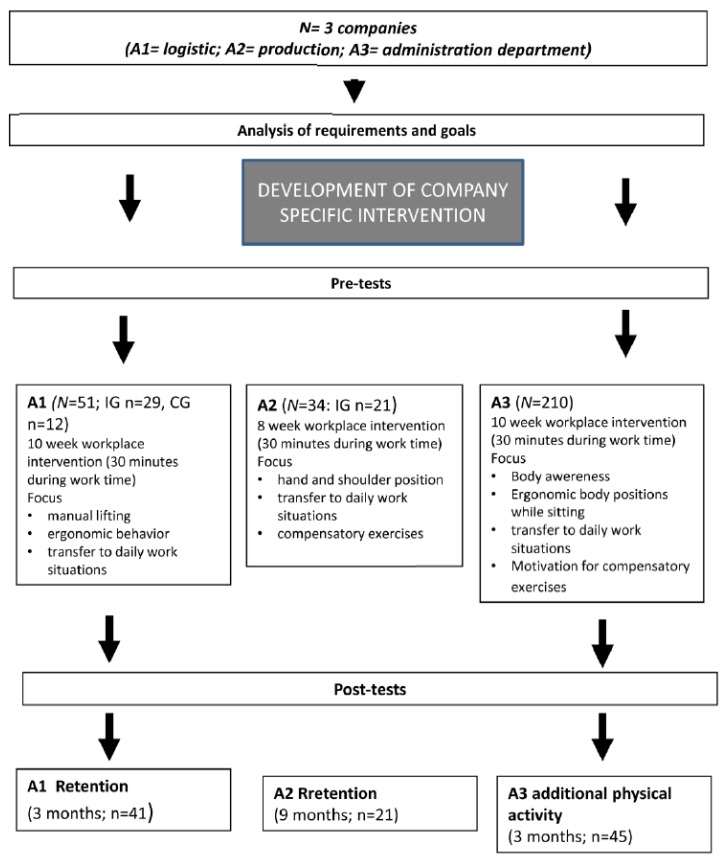
Workflow for all application fields.

Figure 2 gives an overview of all procedures in the application fields.

### 2.2. Participants

#### 2.2.1. Application Field 1

All male logistic workers of a department were examined (*n* = 51: age: 37.5 ± 10.8 years; job tenure: 11.2 ± 8.2 years). Women were excluded because there was only a small group (*n* = 5). Ten males disagreed to take part in the intervention. Depending on the organization processes of the company and the actual availability of the workers, they took part in the intervention group or in the control group which started directly after the first intervention period.

#### 2.2.2. Application Field 2

Altogether 34 employees (only men) aged 38 ± 9.9 years from several departments participated in the investigation. The included sample in Table 1 consists of participants of the three divisions. Participation in this investigation was voluntary. Twenty-one workers were observed for all three measurement points.

#### 2.2.3. Application Field 3

One hundred and seventy-two employees accepted answering the survey. Two hundred and ten people took part in the first intervention, 45 participants went into the second intervention.

Table 1 shows the available demographic data of all participants.

### 2.3. Ethical Statements

All subjects gave their informed consent for inclusion before they participated in the study. The study was conducted in accordance with the Declaration of Helsinki.

### 2.4. Materials and Methods

The assessment of requirements to develop the company specific intervention can be done with several different methods and assessment tools. These are (1) Questionnaires; (2) Observations at the workplace; (3) Physical examinations; and (4) Cognitive representations of movement coordination.

Based on the specific requirements of the companies or the research question, these can be combined or used in isolation.

#### 2.4.1. Questionnaires

Physiological and psychological stresses as well as medical conditions of the employees were evaluated by validated standardized questionnaires [42]. The Slesina Questionnaire [43] revealed information about work conditions (physical, psychological and environmental factors), the intensity of daily work and corresponding individual stress levels. The SF 12 (Short form health survey) analysed physical and mental well-being [44]. The Nordic Questionnaire [42] was used to evaluate work related pain of relevant body areas (e.g., neck, upper body, low back). The Baecke-Questionnaire examined physical activity [45]. 

These standardized instruments were combined with questions that specifically addressed the individual companies’ and employees’ wishes for the interventions (e.g., type of training strength training vs. stress management; ergonomic procedures etc.) and individual barriers to increase health activities. Moreover, we created a feedback procedure with 12 questions about satisfaction with the health promotion interventions. 

#### 2.4.2. Observation of the Workplace

The observations of the workplace were mostly conducted by a team of experts in occupational health in conjunction with employees. The first step was to identify working situations with high impacts on the musculoskeletal system, non-ergonomic conditions and non-ergonomic behaviour of the workers.

These situations were further observed by photo or video analysis with two classification methods to identify objective stressful and straining work situations by two standardized methods: Ovako Working Posture Analysing System [46] and assessment of manual material handling based on Key indicators [47]. Both systems led to the identification of specific task requirement categories, interventions, risk classification and reflection of work related stresses and risks. For specific analysis (e.g., lifting techniques or muscle activity during different daily situations), the risk analysis was combined with biomechanic measurement procedures (e.g., 3D-kinematics or EMG).

Special working techniques (e.g., lifting) were transferred into a standardized observation protocol which included all important stages of the movement (for lifting: body position; hand position in relation to the object; lifting process and relocation of the lifted object).

#### 2.4.3. Physical Examinations

Analysis of physical functions or fitness were executed with different methods (e.g., measurement of the spine (Medi-Mouse^®^, muscle function following Janda [48], heart rate variability or heart rate monitoring, Cavi-Index, body fat analysis etc.). The Progressive Isoinertial Lifting Evaluation (PILE)—Test collected data for lifting processes in a resistance condition [49]. Cardiac stresses, muscle fatigue and lifting quality were analysed. We modified the break off criteria so that the PILE-test ended if: (1) 85% of maximum heart rate was reached; (2) half of the body weight was reached; (3) more than 20 seconds was the time needed for four trials; (4) the employee self-reported muscle fatigue; (5) observed thoracic spine hyper kyphosis; (6) observed lower back hyper lordosis; (7) observed dysfunctional muscle activation; and (8) if the employee self-reported any kind of pain. With criteria (5)–(7), we were able to analyse if the worker lifted in a dysfunctional way. The break-off criteria were observed via video analysis by two physiotherapists independently.

#### 2.4.4. Cognitive Representation of Movement Coordination/Changes in Cognitive Structures

For the logistic and industrial workers, the cognitive representation of ergonomic techniques was assessed by the structural dimension analysis of cognitive representation [50,51,52]. The Basic Action Concepts (BACs) were described to the participants in detail to provide all necessary information to participants similar to other rehabilitation studies [53]. Participants were required to speak the German language fluently. The procedure followed previous research as described by Schack and colleagues [51,52]. Decisions had to be made on impulse, but without any time limits. The whole data acquisition period lasted 15 min.

Table 2 gives an overview of the different surveys and tests which were used in the three application fields.

### 2.5. Statistical Analysis

The collected data was analysed with using SPSS 16.0^©^ (SPSS Inc., Chicago, IL, USA). Common procedures of descriptive statistics (e.g., chi^2^-tests) and frequencies were used. A 3-way ANOVA for the factors group (intervention, control) × weight lifting (kg; treatment parameter for lifting quality) × repeated measurement (Pre, Post) checked a possible interaction (95% confidence interval) in application field 1. For the mental representation (application field 1 and 2), the resulting Euclidian distance matrices formed the basis for a hierarchical cluster analysis (unweighted average linkage) to determine individual representation structures. The cluster analysis is used to reveal structural components within the investigated motor actions. The aim is to identify the mental representation structure implicitly by a splitting procedure of Basic Action Concepts (BAC). Thus, the decision making of participants in the comparison of BACs reveals the underlying representation structure ). 

A last step tested emerged cluster solutions by an invariance measure for structural homogeneity. The statistically suggested threshold to accept invariance is set to a critical value of λ_crit_ = 0.68 (for more details on procedure see Schack et al. [51,52] or for data analysis see Lex et al. [54]). In application field 2, a pre-post-test of the observed working movements was conducted and analysed with chi^2^-tests. The chi^2^-tests were used to test the differences between the pre- and post-test conditions regarding the frequency of the observed ergonomic or non-ergonomic working behaviour of the employees.

## 3. Results

### 3.1. Application Field No. 1—BASE in an International Logistic Company

In cooperation with an international logistic company, BASE was implemented to reduce physical stresses and musculoskeletal complaints by changing work technique behaviour, especially concerning ergonomic lifting. The procedure consisted of three stages.

#### 3.1.1. Implementation of the BASE Concept (Stage One)

The procedure started with the breakdown analysis in cooperation with a company physician and an occupational health and safety specialist.

Physiological and psychological stresses were evaluated using five hours of video material (observing working conditions and workplace stresses of ten employees) which was examined with two classification methods to identify stressful and straining work situations. 

The qualitative video analysis of 10 employees in different departments revealed three common problems of manual lifting processes:

- The organization of the work place, i.e., working height, distance from the object to be lifted and foot and body positions needed to be improved.
- During the lifting process, most of the employees had straight legs with low knee angles which led to stresses on the lower spine.
- Most workers lifted boxes with a thoracic spine hyper lordosis.

These insights provided necessary information in order to design adequate intervention sessions. Data evaluation of the Slesina questionnaire revealed work related stresses. These were a result of heavy manual box handling. In total, 47% of the workers reported holding, lifting and handling of heavy weights as a physical load or stress factors. Psychological stress of concentration (27%) and pressure to perform (20%) were also reported. Participants reported dust, dirt and wind (40%–60%) as environmental stress factors.

Low back pain (lumbar spine 65%), shoulder problems (37%) and neck pain (37%) were the most common musculoskeletal disorders over the last 12 months. Moreover, 28% of the workers had lumbar spine problems during the last seven days. This data reported differences between episodic events of low back pain in the last year compared to acute low back pain of the workers. 

The results of the breakdown analysis were discussed within the observation team and recommendations for interventions were determined. They were separated into proposals for physical, psychological and occupational safety interventions. Environmental changes were implemented by the occupational health and safety department.

#### 3.1.2. Evaluation of Outcome Effects after the Workplace Intervention (Stage Two)

Working conditions with physical stresses were transferred into the workplace intervention. 

Internal organisation issues of the company like shift-work, deadlines and holiday planning disabled a randomized controlled study design. A waiting control group, which started the intervention after the first group had finished, was established. Therefore, logistic workers (*n* = 41) were integrated into a controlled pre-post-test design. The control group got the same intervention after the post-test.

Physical and mental well-being was in the normal range for that age group [44]. 

The intervention group trained box handling techniques during working hours once a week for a duration of 30 min over a period of 10 weeks in small groups of workers. Employees with less than six weeks of participation were excluded. 

Work situations were analysed and cognitive reflected by using the “AHA-experiences”, which gave the employee direct physical feedback of working position, balance or stresses on lower spine. The physical feedback was quickly discussed and dysfunctional movements were identified. Afterwards, the movement task was done again and a new more functional motion sequence was trained. All interventions were accompanied by a feedback procedure about the acceptance of contents, transfer and practicality for daily situations. In addition, workers were taught to coach these concepts, so as to pass their knowledge onto their colleagues.

**Figure 3:**
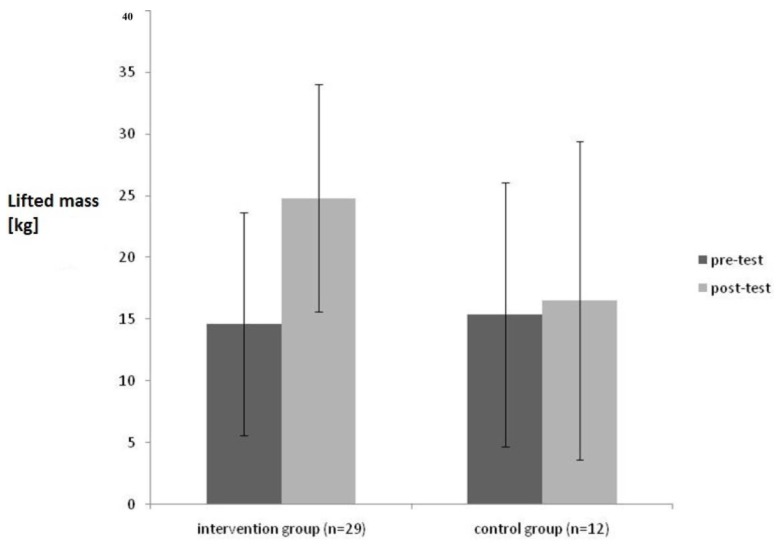
Comparison of the results of the PILE-Test in pre- and post-test condition with (intervention group) and without treatment (control group).

Figure 3 reports the differences between intervention and control group after the intervention.

As shown in Figure 3, there is a significant increase of the lifted weight during the PILE-Test for the intervention group from pre- to post-test (F = 5,51; *p* = 0.02; _p_η^2^ = 1.21), whereas there was no significant change in the control group.

Moreover there was a change in break off criteria for the intervention group.

**Figure 4:**
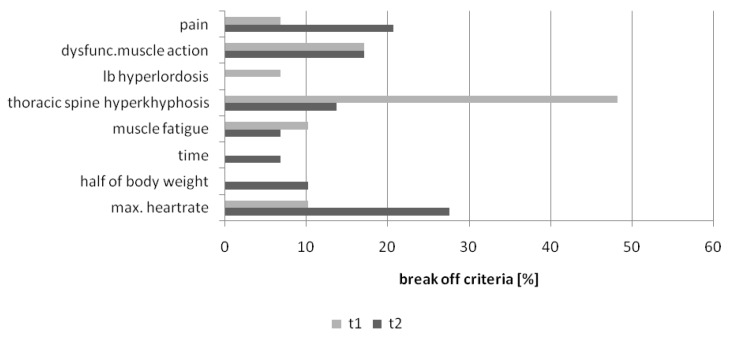
Pre-Post results break off criteria PILE-Test (t1 = pre-test; t2 = post-test) in the intervention group.

As visualized in Figure 4 the number of break offs for “thoracic spine hyper kyphosis” decreased (Χ^2^ = 4.47; *p* = 0.07), whereas the number for the criteria “pain” and “maximum heart rate” and “reaching half of body mass” increased. More participants were able to lift a higher weight with a functional body posture. This resulted in new break off criteria like increasing heart rate (due to more physical work load) and increasing pain (because of lifting weights e.g., 25 kg or higher).

#### 3.1.3. Learning and Lasting Effects of Workers’ Knowledge and Behaviour with Regard to Ergonomic Box Lifting (Stage Three)

**Figure 5:**
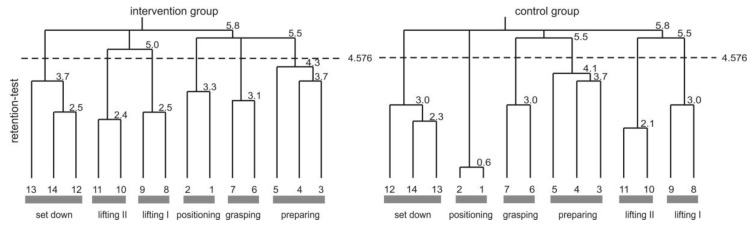
The cognitive representation of the box lifting technique displayed as average cluster solutions of the BACs 1–14 for the intervention and the control group at retention test. The numbers at the bottom represent the used BACs. The Euclidian distances between the BACs is indicated at the conjunctions between BACs. The lower the number at the conjunctions, the lower the Euclidian distances between these BACs. The lower the distance, the stronger the connection between these BACs in long-term memory. The dashed line represents the critical Euclidian distance (dcrit = 4.576 with an error probability of *p* = 0.01) where all branches of the dendrogram were cut-off. BACs connected to one branch below the critical Euclidian distance are clustered. Evolved clusters are indicated by grey bars at the bottom of each dendrogram. The cluster represented functional movement phases named below the grey bars.

The cognitive representation of ergonomic box lifting was assessed by the structural dimension analysis of cognitive representation ([50,51,52] cf. Figure 5). The cognitive representation of the intervention group consisted of five clusters in pre- and post-test (for more details see [10]). In pre-test, their representation was not invariant (λ = 0.49) with regard to a functionally organised representation structure. The evolved functional phases marked the beginning and end of the movement (i.e., *positioning* and *set down*). The remaining clusters did not meet any functional task demands, and revealed a dysfunctional movement structure. The BACs four and six were not integrated in any cluster, and indicated movement problems in this movement phase. The intervention group representation changed throughout the BASE-intervention, and showed a functional optimal organization at post-test (λ = 1.0). Thus, their cognitive representation of box lifting technique reflected perfectly the five functional phases: *positioning*, *preparing*, *grasping*, *lifting*, and *set down*. 

The control groups cognitive representation consisted of four clusters in pre- and of five clusters in post-test. Their cognitive representation was not invariant to a functionally optimal structure, neither in pre-test (λ = 0.57), nor in post-test (λ = 0.50). Nevertheless, representations between pre- and post-test were not invariant to each other (λ = 0.56). In pre-test clusters, the beginning and end of the movement were marked (*positioning and set down*), and the *lifting* cluster was functionally organized. However, the most important BACs (four, six, and seven) do not correspond to any cluster. In post-test BACs, six and seven (*grasping*) were integrated in the cognitive representation leading to a dysfunctional reorganization of the before functionally represented cluster *lifting*. The relevant BAC four was still not integrated in any cluster structure. 

The control group received the identical BASE-intervention as the intervention group before the retention test. It was expected that both groups would show a functional representation of the ergonomic box lifting technique. Both groups got close to, but failed (λ = 0.61) to reach the statistically suggested threshold of λ_crit_ = 0.68 in comparison to the functionally optimal cluster structure. The biggest advancement was the integration of all BACs in cognitive representations, with slight differences in the intra-cluster separation of the *lifting* phase.

### 3.2. Application Field No. 2—BASE in an International Industrial Company 

In cooperation with an international motor tools company, BASE was implemented to enhance sustainable ergonomic movement parameters of a periodic screwing action at the workplace.

The employees produced a whole chain saw in an assembly circuit (cellular manufacturing) under time pressure, because they were paid by the number of the saws produced. 

#### 3.2.1. Implementation of the BASE Concept (Stage One)

Due to a determination of requirements, a survey of stress factors in the daily work routine including potential work related health-risks was applied. This was accompanied by a video analysis of selected working processes in cellular manufacturing, logistic and high storage areas. Special attention was paid to activities with frequent and manual hand movements of heavy loads and the evaluation of the execution of actions in the production circuit. 

In the course of the determination of requirements, a congruence of the symptoms of the employees could be assessed by comparing the results of the Nordic questionnaire with the identified adverse postures from the video analysis. 

The workers reported uncomfortable body positions, holding, lifting and handling of heavy weights as physical load or stress factors. Psychological stress of concentration and attention to detail were also reported. Participants reported dust, dirt and noise as environmental stress factors.

Data from the video analysis pointed out that these health problems can be caused by adverse movement patterns especially from those in the production circuit. The working process in the production circuit is highly specific. Mostly there are unilateral movements with an overuse of the related muscle groups. Furthermore, we discovered improvable working positions especially for the shoulder and hand.

Concerning the storage area, an increased rate of back pain was identified which can be attributed to adverse working positions and adverse postures during lifting and carrying processes as well.

Comparing the different working areas, there were significant differences between the pain referred to the elbows (Χ^2^ = 10.3, *p* = 0.003, *C* = 0.512) and the hand joints (Χ^2^ =7.7, *p* = 0.022, *C* = 0.445)

The determination of requirements resulted from the video sequences of the working processes and the workplace observations on-site as well as from the results of the evaluation of the Nordic and the SLESINA questionnaire. The resultant contents of the program were finally discussed with representatives of the company.

The intervention program is a technical training tool for the handling of manual loads and for the acquisition of compensatory exercises.

It includes:

- Exercises for movement and body awareness inside the working process,
- Reflections of one’s own movement and working behaviour,
- Instructions for movement optimization,
- Instructions for independently executing compensatory exercises, and
- Monitoring of personal health promotion.

The training program focused on the teaching of the principles of movement for load tolerable ways of lifting, carrying, rotating and screwing processes inside the cell production which were basically related to six categories:

1. Workplace organization
2. Positioning in front of loads
3. Optimal way of grabbing
4. Ergonomic screwing operations
5. Ergonomic turning and rotational movements
6. Compensatory exercises for strained muscle groups for home workouts

These principles of movement were addressed and integrated in each training session and were also practiced in different simulated workplace situations.

For the reduction of the mentioned health problems in the production circuit, eight training sessions were designed and conducted within the daily work routine. The identified health problems and areas of pain from the determination of requirements were emphasized and possible causes were reflected on, discussed and clarified via the BASE concept due to “AHA-experience”. Therefore, optimal movements were demonstrated to the employees. From all participants, five intervention groups (IG) were formed. The group formation was affected by rota systems and vacation schedules to allow the selected participants a continuous attendance. The group size was about three to five employees. Each session lasted 30 min and was executed during the shift hours. The concept was completed by an instruction manual for a work-process-oriented muscle strengthening program to strengthen movement resources, to avoid muscular imbalance and to prevent or reduce musculoskeletal disorders.

#### 3.2.2. Evaluation of Outcome Effects after the Workplace Intervention (Stage Two)

To screen the acceptance and for quality management feedback, questionnaires were completed.

Table 3 shows the acceptance and satisfaction with the program.

The interest in the subjects and the acceptance for the training were at a high level. Thus, the overall feedback for the intervention was very positive. A vast majority of the trained employees could establish a direct link to their own workplace. Moreover, the exercises were considered useful.

After completing the training, every employee got a Thera-band and an exercise catalogue for self-organized practice at home which was tailored for the specific muscles used in the working process.

To prove the sustainability of the implemented optimal movements in the daily work routine, an evaluation was conducted six months later. 

The pre-post monitoring mentioned that all training contents were still implemented in the working process at the point of evaluation.

#### 3.2.3. Learning and Lasting Effects of Workers’ Cognitive Representation and Behaviour of an Ergonomic Screwing Action (Stage Three)

To evaluate the lasting effects of the intervention, (1) the movement outcome was analysed by experienced physiotherapists; and (2) the cognitive representation was analysed to verify changes in the memory structures regarding movement organisation. 

Table 4 shows the aggregated results of the evaluation of workers’ ergonomic behaviour by experienced physiotherapists.

The results showed that workers executed a more ergonomic working behaviour during the screwing movement in comparison to the pre-post conditions. The descriptive data analysis shows generally that the percentage of workers executing an ergonomic behaviour increased significantly from pre- to post-intervention (Table 4). However, only the body segments shoulder (BAC 7 and BAC 10) as well as trunk (BAC 4 and BAC 11) show slight positive to negative changes after the intervention.

**Figure 6:**
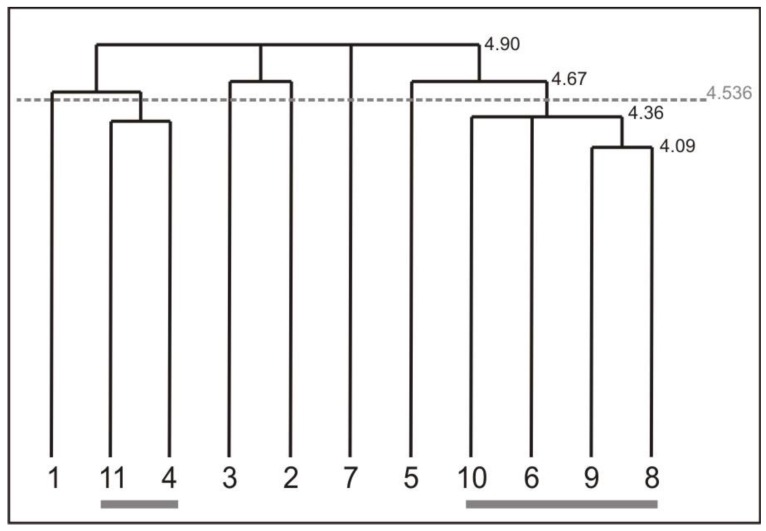
Mean results of the cognitive representation of the screwing action of all workers measured at the retention test. For interpretation of the dendrogram, see Figure 5 in section 3.1.3. The used BACs are labelled in Table 4.

Figure 6 shows the mean results of the cognitive representation of the screwing action of all workers measured at the retention test. Overall, the representation structure shows a tendency to differentiate between different body segments. Two clusters evolved during the intervention of the workers repeatedly executing the screwing action. Cluster one is formed by the BAC 4 (trunk in upright position/ straight back) and BAC 11 (back stays straight). Both BACs indicate body segments connected to the trunk, which are closely related to foot position (BAC 1). Cluster two is formed by BAC 6 (arms near the trunk/ flexed), BAC 8 (wrist joint straight and fixed), BAC 9 (elbow stays near the trunk), and BAC 10 (shoulders stay in a lowered position). Basically, all can be associated to the connection of the shoulder, arm, elbow, and wrist to execute a movement correctly and grasp the tool properly (BAC 5). In addition, BAC 2 (knee) and BAC 3 (hip) are loosely connected to each other. However BAC 7, the active process of *shoulders lowered* is singled out in workers cognitive representation. This is one of the most important processes for ergonomic movement execution, and should be integrated in cluster two. This confirms the finding of the analysis of the movement outcome. The physiotherapists confirmed that less advances are made by the workers regarding ergonomic shoulder actions, which is confirmed by the representation structure. Thus, not all BACs were sustained in workers’ memories during the eight week intervention.

### 3.3. Application Field No. 3—BASE in Office Workers in a Management Department

The health management department at the University of Hamburg tried to identify global psychological, physical and environmental stresses of employees. Recent analysis of special working conditions and following interventions in two pilot projects failed to maintain acceptance. They were seen as superficial and not goal orientated by the employees. Therefore, the BASE concept was chosen to satisfy and motivate the office workers to participate in additional physical activity.

#### 3.3.1. Implementation of the BASE Concept (Stage One)

In the first steps, all requirements for health prevention (psychological, physical and environmental stresses and strains) were examined. The results were categorized into the three domains (1) psychological; (2) physical; and (3) environmental stress and afterwards presented as an overview of evaluated departments. 

Time pressure, deadlines and pressure to perform were the common psychological strains (40%–50% of the workers reported these conditions). Physical stresses were related to sedentary workstyle (sitting; >80%) and uncomfortable, forced body positions (>50%). Uncomfortable environmental factors include attention to detail, IT problems and reliance on the unfinished work of colleagues (>40%). These work related strains were accompanied by neck, shoulder and back pain.

The results were presented and discussed with the occupational health and safety team, other members of health management and the heads of involved departments. Afterwards, each professional area developed interventions for their domains in order to meet the requirements of the corresponding department. In addition, the workers were asked about their specific wishes for the topics of the interventions. The main wishes were movement exercises and ergonomic lessons at the workplace (70%).

#### 3.3.2. Evaluation of Outcome Effects after the Workplace Intervention (Stage Two)

Based on the results of workplace requirements, the BASE intervention was conducted over 10 sessions for 30 min each at the workplace in small groups (5–8 persons).

The sessions included:

- Exercises to be aware of discomfort body positions
- Working height and sitting position
- Workplace and work organisation
- Dynamic sitting
- Detail seeing and the consequences of the head position to reduce neck pain
- Shoulder flexibility
- Ergonomic mouse handling
- Motivation for additional physical activity

The acceptance of the program was evaluated with the feedback questionnaire (cf. Table 5.).

#### 3.3.3. Motivation and Satisfaction with Additional Exercises (Stage Three)

The third step in this field was to motivate the office workers to take part in additional exercises, with the same duration as the BASE intervention. Only a few office workers (*n* = 45) were able to manage this additional physical activity. The evaluation of this stage was also used to get some information about the conditions e.g., barriers for ongoing physical activities (Table 6). 

All interventions were highly accepted by the employees. Especially, the additional physical exercise was successful and benefited physical and mental well-being. Moreover, the participants asked for ongoing physical activity during working hours. One important barrier was the fact that not all supervisors allowed their employees to join the intervention during working hours.

## 4. Discussion

### 4.1. Implementation of the BASE-Program in the Three Application Fields (Stage One)

The BASE-program which was created to prevent musculoskeletal disorders was implemented in three different fields. The process of enhancing health promotion was adapted to the requirements of the involved companies to achieve greater compliance and positive results for future health related interventions.

In the first of four consecutive steps, all requirements for health prevention (psychological, physical and environmental stresses and strains) were examined with a combination of different suitable methods. The results have been categorized into three domains (1) psychological; (2) physical; and (3) environmental stress, and afterwards presented as an overview for the evaluated departments. One advantage of the combination is that there will be detailed information about stress factors in all three domains. This information can be used to plan directed interventions. When comparing the three applications fields, it is obvious that each working condition has its own specific risk profile with resulting stresses or pain in different musculoskeletal areas. Our experience has shown that standardized interventions like classic back schools are mostly not able to integrate all aspects we analysed to be important. In line with Marshall [21], we found that a successful program needs to meet the needs of employees. 

The results were presented and discussed with occupational health and safety personnel, other members of the health management team and the heads of involved departments. This was useful in gaining compliance among involved supervisors and stakeholders.

The BASE-program integrates all phases which Hammer et al. [55] proposed for adherence to WHPP. They suggested four phases which develop strategies to increase adherence to WHPP at the personal and organizational level. Phase one has to identify health hazards [55]. This was done in the BASE-program with the analysis of requirements. The results were implemented in the interventions which then included multicomponent programs, environmental and individual approaches, educational advertisement and consideration of the organizational climate as suggested by Hammer et al. [55] in phase two. Afterwards, the implementation phase of the training took place. It included engaging and motivating actions. Hammer et al. [55] appointed phase four: maintaining and providing a positive relationship between management and workers as well as facilitating self-efficacy. In contrast to Hammer, this phase was implemented from the initial stage of the BASE-program, because it was shown by Menzel et al. [56] that the promotion of health prevention by the supervisors is one key aspect in running a successful program. Supervisors can communicate and promote the activities and if they join the activities first they may be act as a role model for health behaviour.

Overall, the concrete analysis of the health related problems for examined working places, the exchange of information between the involved persons, participation of the employees and the adapted training or intervention are relevant factors for success. In addition, it has been shown, that tailored exercise programs concerning the individual pain and movement decrements of the workers are suitable to reduce pain symptoms (e.g., neck and upper limbs [57,58]). Moreover, these programs sufficiently increased the flexibility [57,58]. These effects can only be reached by an extensive breakdown analysis. 

### 4.2. Evaluation of the Outcome Effects of the Interventions (Stage Two)

All reported interventions were problem-orientated and based on participation to determine compliance. The BASE intervention integrates coordination, perception and technique. Workers learn to be aware of their body positions during the working process to be able to correct dysfunctional body positions. The employees’ own experience and perceptions is a useful key to reflect on individual behaviour. These points were focussed on everyday work situations, while the exercises were arranged to fit their working place. The different natures of the working conditions as well as the different risk profiles and requirements required specific adaptions for the three separate application fields. Moreover, for each application field, different outcome measurements have to be used (due to the goal of the intervention or to the acceptance of the company’s management). Therefore, the outcome effects will be discussed separately.

#### 4.2.1. Application Field 1 Logistic Workers

In application field 1, the results of the PILE-test have shown that a technique orientated education of the box-lifting process might reduce low back pain and prevent musculoskeletal disorders. Under stress conditions during the PILE-test, it was obvious that low technical experience resulted in dysfunctional behavior. The problem orientated work place intervention decreased dysfunctional lifting behavior during the PILE-test, which resulted in an increased lifted weight and a change in break off criteria. 

As reported, there was an increase of pain for some of the workers during the test. This might be a result of the individual’s physical conditions (self-reported musculoskeletal disorders) in combination with the increased weight during the test. 

The cognitive representation of a box lifting technique of workers allows a conclusion on the actual movement outcome. The ergonomically relevant Basic Action Concepts four (back straight) and six (functional grasping) were integrated in workers’ cognitive representation after the BASE-intervention. These concepts built key factors for a successful intervention in training a box lifting technique. 

Nevertheless, a study limitation is that the performance of the control group was not a result of a direction of a functional movement organization, instead they changed, and the reason remains unclear. It is possible that colleagues (who first participated in the intervention) provided hints regarding the interventions to them, and they started training these skills by themselves. It is also possible that the workers remembered instructions and tips of former interventions they participated in. Thus, they became more sensitive to ergonomic box lifting. Whatever the cause of their improvements, they still remained far behind the results from the BASE-intervention group. 

We included the recommendation of Martimo et al. [59] that work related interventions for handling techniques will only be successful if they integrate participating aspects. We suggest that this was one reason for finding positive effects and gaining general acceptance by the workers for the program. It is concluded that, especially this group of employees, with little motivation to participate in resistance training interventions in their spare time, benefit from the methodological approach of this intervention.

#### 4.2.2. Application Field 2 Cellular Manufacturing

The implemented training program in application field 2 combined measures of situational and behavioural prevention in three ways. First, the present gap between the offered health-promoting programs and its execution in the daily work routine in the course of the intervention was noted and the problems were presented. Furthermore, the intervention focused on training the conscious perception of the own body positions while lifting, carrying, rotating and screwing processes and the question was how to avoid stressful body positions by using minimal change of motion, optimized working conditions and work organization with an increased use of tools. 

As observed in application field 1, a great amount of acceptance was gained. However, there were some concerns by the employees that the duration of the intervention was too short. Only 33% agreed with the duration of the exercises. Moreover, in contrast to the intervention in application field 1, there were additional exercises related to muscle strengthening in this group. These exercises had to be completed in the 30 min session as well as the technique orientated tasks. We suppose that this was not a sufficient time frame. The intervention was two weeks shorter than the intervention of Application field 1 and integrated more aspects of motor learning and physical training.

#### 4.2.3. Application Field 3 Office Workers

As shown in application field 1 and 2, the intervention was adapted to meet the requirements and specific wishes of the employees. This resulted again in acceptance of the intervention by the workers. The intervention was rated as useful and the participants had fun during the sessions. Following Roepke et al. [60], the individuals’ views on health promotion have an impact on positive health behavior. In addition, DeSteno et al. [61] suggested that emotions have a high impact on health behavior. We suppose that our intervention enhanced positive meanings and emotions related to health promotion. Unfortunately, we did not control for this. Menzel et al. [56] also supports this idea and analyzed the main motivation for participants (*n* = 339) engaging in a WHPP. Participants report that they wanted to improve their fitness levels, well-being and, in addition, 41.7% of the employees wanted the intervention to be fun.

### 4.3. Lasting Effects and Enhancing Health Promotion (Stage Three)

All examples of the different working areas showed sustained effects on health promotion, evaluated by different outcome measurements.

#### 4.3.1. Application Field 1 Logistic Workers

The retention test results showed a tendency to adapt the functionally optimal representation of the ergonomic box lifting technique in both groups. Two conclusions can be inferred from these results. Firstly, the functionally optimal representation of the intervention group is sustained for a longer time period. Hence, the BASE-intervention can be evaluated as a well suited tool to change behavior for an extended period of time. Secondly, the functionally optimal representation of the control group (which received BASE-intervention between post- and retention-test) confirms the results from the intervention group. 

The results revealed that the interventions in the BASE-program are able to influence the cognitive representation of a box lifting technique and therefore positively influence the movement outcome. The workers who participated in the BASE-intervention showed a more ergonomic movement initiated by a more functional cognitive representation in long term memory. Therefore, the BASE concept can be described as an intervention which reduces work related physical stress factors successfully, being both enduring and effective. However, future research is needed to control the study effects with a RCT.

#### 4.3.2. Application Field 2 Cellular Manufacturing

As observed in Table 4, the intervention resulted in lasting effects of changes in body positions to increased ergonomic working behaviour. However, employees struggled to adapt a load compatible position of the shoulders. Both the movement observation and the evaluation of the structure of cognitive representation of the analysed screwing operations gave evidence to suggest that inappropriate positions of the shoulder are captured already at time of the preparation for the production step and is not corrected in the whole sequence. 

The analysis shows that the adverse shoulder positions are a result of particular handling of the working tool. Due to the attachment (power source of the screwdriver), it has to be reached from above. After grabbing the tool, the shoulder remains in an upward position and is not placed back in the starting position. This observation was also pointed out within the results of the cognitive representation. In this case, the shoulder position remains isolated from all other movements. Therefore, there is still a need to practice the load tolerable shoulder posture.

Nevertheless, the given compensation program was perceived as helpful but in fact only a few employees continued with the exercises after the intervention ended. In the course of the training program, a fundamental basis for the initiation of sustainable changes in movement behaviour and personal responsibility was established. Focused characteristics such as balance, upright trunk body alignment with a straight back, as well as the hand positions during work processes were integrated into the daily work routine even nine month after the program. Besides this, awareness and personal interest of the employees for health-promoting measures arose. 

#### 4.3.3. Application Field 3 Office Workers

The additional exercise intervention with the office workers was able to gain the satisfaction of the workers. Moreover, the workers reported that the intervention improved their physical (92%) and mental (84%) well-being. In addition, the intervention was rated as useful and the health related contents were liked by the workers. According to Proper et al. [22] who stated that the implementation quality is of particular importance for the success of WHPP, we tried to manage this criterion by developing a suitable intervention. This was done again by referring to the results of the requirement analysis. Unfortunately, only 45 employees were able to join the intervention. The main barrier was that the supervisors did not support the second intervention, although the supervisors believed in the benefits of the first intervention. One possible explanation for this reaction might be the notion that health insurance programs in Germany state that health promotion interventions should last 8–12 weeks and after the program the participants are responsible for their ongoing health behavior. Another point is that the employees agreed to the program in their working hours and most of them would pay for it, but only 30% would do an intervention after work. This supports the idea that own health promotion at the office did not reach the status of an automatized behavior. Due to the fact that forming a new habit needs a specific amount of time ([62] found an average of 66 days), one might think about supervisors agreeing to increase the duration of interventions for future health promotion programs.

## 5. Limitations

Due to the conditions of the participating companies and the structures of the examined departments, we were not able to calculate sample sizes. However, this is one major problem in WHPP or OHP studies. Nevertheless, there might have been more powerful study results if the study design could have been done as a randomized controlled trial with a priori calculated sample size. 

## 6. Conclusions

The implementation of the BASE-concept in all reported application fields was greatly approved and accepted by all involved participants. The feedback of the employees suggests that they might have a strong motivation for a continuous performance of the exercises and interventions. 

Non-ergonomic working positions have been sustainably reduced (in application fields 1 and 2) with lasting effects. Moreover, the BASE-program was suitable in increasing motivation for additional physical activity. 

According to our project experience, the following aspects should be integrated to continue the development of WHHP:

- The employees should be involved actively in the program to express and comment on their needs and to clarify questions of understanding.
- Further instruments are needed (e.g., multipliers, posters, flyer, homework materials), to establish steady functional memory structures within each employee.
- Just the exercise program for the home workout is not sufficient for an obligatory performance of the exercises. In this case, a link to company sports or other prevention courses should be considered.
- The intervention programs should be tailored and individualized.
- Referring to change automatized movements or habits, the interventions should include more practice and transfer units within different working conditions and situations.

All mentioned aspects can optimize the initiated problem-related and practice-based prevention activities. As shown with the three different application fields, the presented structure of the BASE-program can be transferred to other divisions without any difficulty.

## Figures and Tables

**Table 1:** Overview of participants all application fields.

Participants	Age (Years)		Body Height (cm)		Body Mass (kg)		Affiliation (Years)	
Application Field 1 (*n* = 51)	Mean	SD	Mean	SD	Mean	SD	Mean	SD
Intervention (*n* = 29)	36.0	11.7	176.6	7.6	83.5	12.3	9.4	7.9
Control (*n* = 12)	39.6	9.9	176.3	7.6	83.3	13.4	13.0	8.5
Application Field 2 (*n* =34)	38.9	9.9	176	8.8	81.2	13.8	8.1	9.75
Application Field 3 (*n* = 174)								
Woman (*n* = 132)	42.9	11.4	168.7	6.9	68.9	15.6	n.a	
Men (*n* = 32)	45.3	10.6	181.8	7.3	77.1	12.8	n.a	

**Table 2:** Methods used in the different application fields.

	Application Field 1 Logistic Workers	Application Field 2 Industry Workers	Application Field 3 Office Workers
Analysis of requirements	Slesina Questionnaire	Slesina Questionnaire	Slesina Questionnaire
	Nordic questionnaire	Nordic questionnaire	Nordic questionnaire
	OWAS	OWAS	OWAS
	Key Indicators	Key Indicators	Key Indicators
	SF12	SF12	SF12
	Muscle function	Cognitive representation	
	Cognitive representation		
Pre-post analysis	Slesina Questionnaire	Movement analysis	Feedback (acceptance of intervention)
	Nordic questionnaire	Cognitive representation	
	PILE-Test	Feedback (acceptance)	
	Cognitive representation		
	Feedback (acceptance)		
Retention analysis	PILE-Test Cognitive representation	Movement analysis	Feedback (acceptance and outcomes of ongoing physical activity)
		Cognitive representation	
		Feedback (acceptance)	

**Table 3:** Satisfaction with the intervention Application field 2 (*n* = 34).

Number of Feedback Responses for All Intervention Sessions (*n* = 143)	Yes, I Totally Agree	I Agree	No, I Disagree
Questions	(%)	(%)	(%)
1. I liked the exercises.	57	29	14
2. The exercises hindered my work.	18	33	49
3. I think that the exercises are useful.	64	26	10
4. Today I learnt something new.	58	27	15
5. I remembered previous teaching of ergonomic behaviour.	20	38	42
6. I had fun during the exercises.	52	36	12
7. The exercises beared reference to my daily working situations.	51	36	13
8. I was able to execute the exercises.	50	38	12
9. The trainers explanations were clear and comprehensible.	72	19	9
10. I was satisfied with the support I received.	72	20	8
11. The duration of the exercises was suitable.	33	46	21
12. I would like to get more information about health promotion.	60	28	12

**Table 4:** Movement Analysis (*n* = 21 pre-retention-test).

Movement Phase/Cluster	Body Segment	Ergonomic Criteria of the BAC	Execution beforeIntervention (%)			Execution 9 MonthPost Intervention (%)			Chi^2^*p*-Value
			No	Partly	Yes	No	Partly	Yes	
Body position at the work space 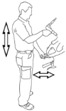	Foot positon	BAC 1: feet parallel and shoulder width apart/whole foot on the floor	0	82	18	0	27	73	60.98
									<0.001
	knee	BAC 2: knee slightly bent	34	64	0	18	36	46	52.42
									<0.001
	hip	BAC 3: hip centred over BoS	0	91	9	0	64	36	20.9
									0.033
	trunk	BAC 4: trunk in upright position/ straight back	0	55	45	9	36	55	not significant
Grip position of the working tool 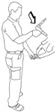	hands	BAC 5: grip position according to the working tool	0	64	37	0	18	82	42.82
									<0.001
	arms	BAC 6: arms near the trunk/flexed	0	73	27	0	18	82	60.94
									<0.001
	shoulders	BAC 7: shoulders lowered	36	55	19	9	64	27	n.s.
Working activity 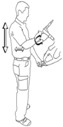	hands	BAC 8: wrist joint straight and fixed	36	64	0	0	18	82	104.98
									<0.001
	elbow	BAC 9: elbow stays near the trunk	46	46	8	0	9	91	91.12
									<0.001
	shoulder	BAC 10: shoulders stay in a lowered position	64	27	9	18	82	0	22.19
									<0.001
	trunk	BAC 11: back stays straight	9	55	36	9	55	36	not significant

**Table 5:** Satisfaction with the intervention (*n* = 174).

Number of Feedback Responses for All Intervention Sessions (*n* = 1112)	Yes, I Totally Agree	I Agree	No, I Disagree
Questions	(%)	(%)	(%)
1. I liked the exercises.	84	15	1
2. The exercises hindered my work.	26	38	37
3. I think that the exercises are useful.	92	7	1
4. Today I learnt something new.	72	24	4
5. I remembered previous teaching of ergonomic behavior.	39	39	22
6. I had fun during the exercises.	74	24	2
7. The exercises bore reference to my daily working situations.	88	12	0
8. I was able to execute the exercises.	93	6	1
9. The trainers’ explanations were clear and comprehensible.	96	2	2
10. I was satisfied with the support I received.	72	20	8
11. The duration of the exercises was suitable.	51	40	9
12. I would like to get more information about health promotion.	55	35	10

**Table 6:** Feedback after additional exercise at the workplace (10 sessions).

Number of Feedback Responses (*n* = 37)	Yes, I Totally Agree	I Agree	No, I Disagree
Questions	(%)	(%)	(%)
1. I liked the exercises.	95	5	0
2. I liked the health education during the exercises.	95	5	0
3. I think that the physical exercises are useful.	100	0	0
4.The intervention improved my physical well-being	92	8	0
5. The intervention improved my mental well-being	84	16	0
6. I would like to take part in ongoing exercises at the workplace.	92	8	0
7. The exercises beared reference to my daily working situations.	84	16	0
8. I was able to execute the exercises.	92	8	
9. The trainer explanations were clear and comprehensible.	97	3	2
10. I would only take part in ongoing exercises if there are no fees.	41	38	16
11. I would take part in ongoing exercises if there are fees (e.g., 25 Euro for 10 sessions).	32	46	22
12. I would take part in ongoing exercises after working hours.	30	40	30
